# Modulation of defence and iron homeostasis genes in rice roots by the diazotrophic endophyte *Herbaspirillum seropedicae*

**DOI:** 10.1038/s41598-019-45866-w

**Published:** 2019-07-22

**Authors:** Liziane Cristina Campos Brusamarello-Santos, Dayane Alberton, Glaucio Valdameri, Doumit Camilios-Neto, Rafael Covre, Katia de Paiva Lopes, Michelle Zibetti Tadra-Sfeir, Helisson Faoro, Rose Adele Monteiro, Adriano Barbosa-Silva, William John Broughton, Fabio Oliveira Pedrosa, Roseli Wassem, Emanuel Maltempi de Souza

**Affiliations:** 1 Department of Biochemistry and Molecular Biology, Federal University of Parana, Curitiba, PR Brazil; 2 Department of Clinical Analysis, Federal University of Parana, Curitiba, PR Brazil; 3 Sector of Professional and Technological Education, Federal University of Parana, Curitiba, PR Brazil; 4 Federal Institute of Materials Research and Testing, Division 4 Environment, Berlin, Germany; 5 Department of Genetics, Federal University of Parana, Curitiba, PR Brazil; 6 Present Address: Department of Biochemistry and Biotechnology, State University of Londrina, Londrina, PR Brazil; 7 Present Address: Department of Neuroscience, Icahn School of Medicine at Mount Sinai, New York, USA; 8 Present Address: Carlos Chagas Institut – Fiocruz, Curitiba, PR Brazil; 9 Present Address: Luxembourg Centre for Systems Biomedicine (LCSB), University of Luxembourg, Esch-sur-Alzette, Luxembourg

**Keywords:** Gene expression analysis, Transcriptomics

## Abstract

Rice is staple food of nearly half the world’s population. Rice yields must therefore increase to feed ever larger populations. By colonising rice and other plants, *Herbaspirillum* spp. stimulate plant growth and productivity. However the molecular factors involved are largely unknown. To further explore this interaction, the transcription profiles of Nipponbare rice roots inoculated with *Herbaspirillum seropedicae* were determined by RNA-seq. Mapping the 104 million reads against the *Oryza sativa* cv. Nipponbare genome produced 65 million unique mapped reads that represented 13,840 transcripts each with at least two-times coverage. About 7.4% (1,014) genes were differentially regulated and of these 255 changed expression levels more than two times. Several of the repressed genes encoded proteins related to plant defence (e.g. a putative probenazole inducible protein), plant disease resistance as well as enzymes involved in flavonoid and isoprenoid synthesis. Genes related to the synthesis and efflux of phytosiderophores (PS) and transport of PS-iron complexes were induced by the bacteria. These data suggest that the bacterium represses the rice defence system while concomitantly activating iron uptake. Transcripts of *H*. *seropedicae* were also detected amongst which transcripts of genes involved in nitrogen fixation, cell motility and cell wall synthesis were the most expressed.

## Introduction

To answer the ever increasing demand for cereals, genetic improvement of rice and the concomitant development of bio-fertilisers are promising, low environmental-impact solutions. After water, the most limiting nutrient in plant development is nitrogen and nitrogenous fertilisers have been heavily used in rice cultivation^1^. Heavy use of nitrogen fertilisers causes environmental damage including contamination of ground-water and the release of nitrogen oxides.

An alternative to the use of nitrogenous fertilisers is to employ plant-associated micro-organisms that fix nitrogen. *Herbaspirillum seropedicae* is an endophytic diazotroph that can colonise many plants and improve their productivity (reviewed by Monteiro *et al*. and Chubatsu *et al*.^2,3^). Inoculation of rice with *H*. *seropedicae* increased root and shoot biomass by 38 to 54% and 22 to 50% respectively^4^ part of which was attributable to biological nitrogen fixation^4–7^. Pankievicz *et al*.^8^ showed that the nitrogen fixed by *H*. *seropedicae* and *Azospirillum brasilense* was rapidly incorporated into *Setaria viridis*. Other factors, including production of phyto-hormones by the bacteria stimulate plant growth and several authors have observed that the increase in biomass of inoculated plants is dependent on the plant genotype^4,9^.

Transcriptome based studies are powerful tools to detect differentially expressed genes (DEG) and discover novel molecular processes^10–16^. Expression analyses (using EST sequencing and RT-qPCR) of rice roots inoculated with *H*. *seropedicae* suggested that genes related to auxin and ethylene syntheses as well as defence are modulated by the microorganism in a cultivar dependent manner^17^. Therefore, the purpose of the present study was to determine the effect of *H*. *seropedicae* on the gene expression of rice roots by RNA-seq transcriptome analyses. The data showed a genome wide repression of plant defence genes and activation of iron uptake systems, which seems an important feature for successful root colonization.

## Results and Discussion

### Transcriptional analyses

*H*. *seropedicae* enters rice roots via cracks at the points of lateral root emergence and later (three to 15 days) colonises the intercellular spaces, aerenchyma, cortical cells and vascular tissue^4,6,7,18^. 14 days after inoculation we observed an increase of weight of roots and leaf but this increase was not statistically significant. The number of endophytic *H*. *seropedicae* reached approximately 10^5^ to 10^6^ CFU per gram of fresh root weight one to two days after inoculation (DAI), with a peak at three DAI (Supplementary Fig. 1). For this reason roots were collected for RNA-seq analyses three DAI when the population of *H*. *seropedicae* had stabilised in the intercellular spaces and xylem^7^. Rice plants inoculated in parallel with the samples used for RNA extraction contained 4.2 × 10^5^ endophytic CFU.g^−1^ of fresh roots and 4.4 × 10^8^ epiphytic CFU.g^−1^ of root at three DAI.

Sixty-four percent of the reads (103,563,118) were finally used for mapping to the reference rice and *H*. *seropedicae* genomes. Illustration of RNA-seq analyses is shown in Supplementary Fig. 2 and the numbers of reads mapped to each reference genome are listed in Supplementary Table 1. Mapping on the rice genome database (http://rice.plantbiology.msu.edu/) produced 22 million unique mapped reads representing 13,837 expressed transcripts.

### Differentially expressed genes

**Figure 1:**
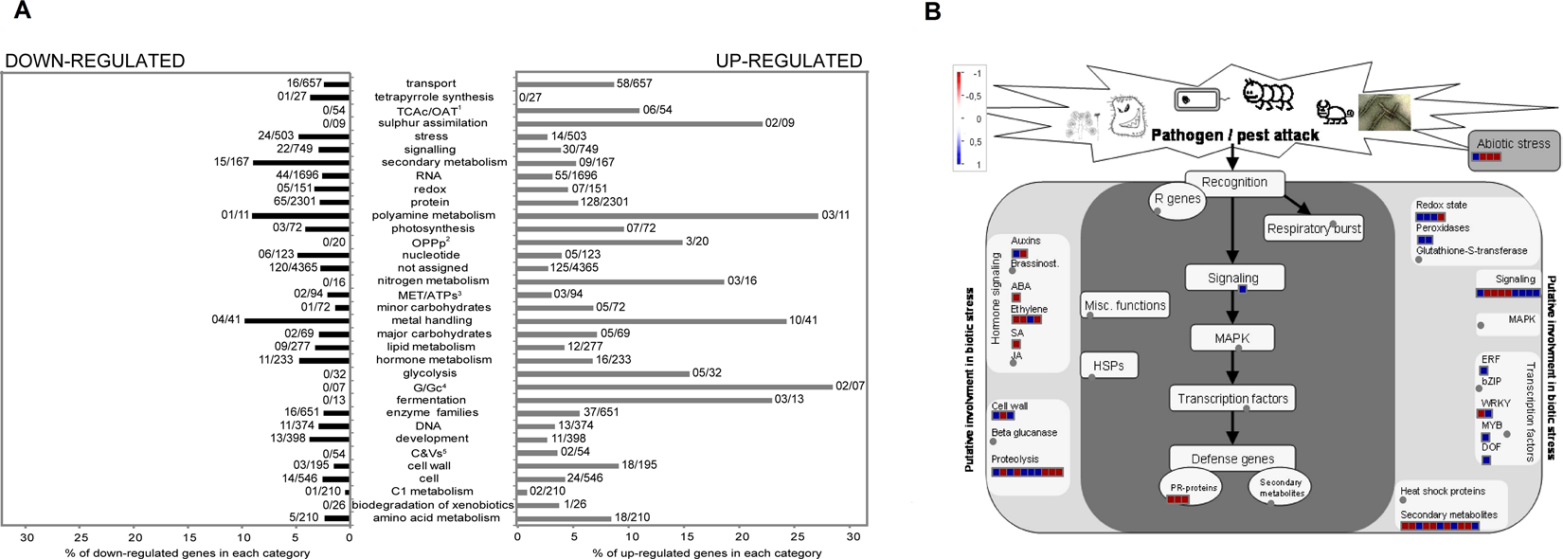
Transcripts differentially expressed in rice roots colonised by *H*. *seropedicae* were grouped according to metabolic categories according to MapMan. Overview metabolism is shown in Panel A and biotic and abiotic stress in Panel B. **(A)** Up-regulated genes are shown in the right-hand column (in gray) and down-regulated genes in the left column (in black). Numbers of regulated genes and total numbers of expressed genes are shown for each category. ^1^TCA cycle/organic acids: ^2^oxidative pentose phosphate pathway; ^3^mitochondrial electron transport/ATP synthesis; ^4^gluconeogenesis/glyoxylate cycle; ^5^cofactor and vitamin synthesis. **(B)** The scheme was constructed using only genes with fold change ≥2; ≤2. Small squares represent up-regulated (blue) or down-regulated (red) genes.

Statistical analyses were performed using DESeq software^19^ and comparison of non-inoculated with inoculated samples revealed 1,014 differentially expressed transcripts (P < 0.05) (Supplementary Table 2), with 255 having fold-changes higher than two. A heat map constructed using the expression profiles of these transcripts showed the inoculated sample clustering clearly separated from the control samples (Supplementary Fig. 3). In addition, functional categorisation of these set of genes was performed using MapMan (http://mapman.gabipd.org/) (Fig. 1B) and Blast2GO^20^ followed by GO enrichment with Gene Set Enrichment Analysis (GSEA)^21^. In the Biological Process category the following GOs terms were enriched: homeostatic process, drug metabolic process, carboxylic acid metabolic process, transmembrane transport, ion transport, response to chemical, regulation of transcription, DNA-templated, oxidation-reduction process and small molecule biosynthetic process (Supplementary Table 3).

MapMan analyses also revealed several rice roots pathways regulated by *H*. *seropedicae* colonisation. Considering the number of regulated genes in relation to the number of expressed genes in each category, the main MapMan categories down-regulated by *H*. *seropedicae* were: metal handling (9.0%; 4/41), polyamine metabolism (9.1%; 1/11), secondary metabolism (9%; 15/167), hormone metabolism (4.7%; 11/233); stress (4.8%; 24/503) and nucleotide metabolism (4.9%; 6/123). Among genes up-regulated by bacteria were those involved in gluconeogenesis/glyoxylate cycles (28.6%; 02/07), polyamine metabolism (27.3%; 03/11), metal handling (24.4%; 10/41), fermentation (23.1%; 03/13) and nitrogen metabolism (18.8%; 03/16) (Fig. 1A). Since MapMan was specifically developed to cover plant-specific pathways and is widely used by plant researchers, some of genes of these regulated categories are analysed in more detail below.

### Biotic and abiotic stresses

Among the 255 differentially expressed genes (>2fold), 59 were stress-related (30 repressed and 29 induced) in the following categories: secondary metabolites, hormone signalling, cell-wall, proteolysis, PR-proteins, signalling, transcription factors, redox-state, abiotic stress and peroxidases (Fig. 1B and Supplementary Table 4).

#### Secondary metabolism

**Figure 2:**
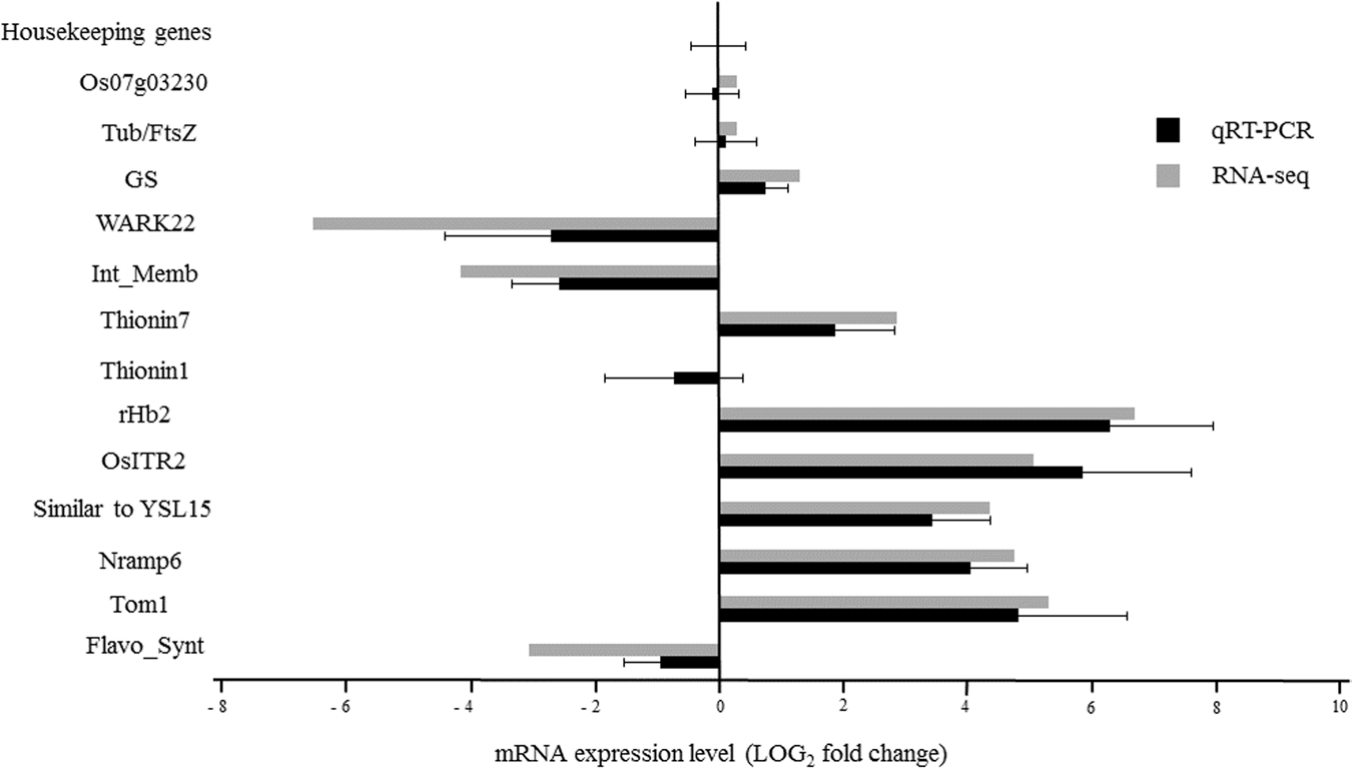
Confirmation of differential expression of rice genes by qRT-PCR and RNA-Seq. The results are average of three independent samples and error bars represent, the standard deviation. The reference genes used for the analysis were actin 1, tubulin beta-2 chain and conserved hypothetical protein.

Amongst the secondary metabolic pathways with higher numbers of regulated genes were those involved in phenylpropanoid and isoprenoid synthesis (Supplementary Table 4). Phenylpropanoids synthesised by deamination of L-phenylalanine are eventually converted to *p*-coumaric acid, a precursor of flavonoids and lignin^22,23^. Additionally, *H*. *seropedicae* modulated expression of four genes involved in flavonoid synthesis. Amongst them, the gene encoding chalcone isomerase (CHI), which catalyses the synthesis of naringenin from tetrahydroxy-chalcone^24^, was repressed 2.1-fold. Naringenin is a key intermediate in the synthesis of other compounds including flavonol [flavonol synthase (FS), repressed 8.3-fold by *H*. *seropedicae*], and anthocyanins [dihydroxiflavonol 4-reductase (DRF), repressed 2.5-fold]. In addition, isoflavone reductase (IFR) transcript, involved in isoflavone synthesis, was repressed 1.3-fold (P = 0.01). RT-qPCR of FS confirmed repression by *H*. *seropedicae* (Fig. 2) but to a much lower extent (1.8-fold). Previously, Balsanelli *et al*.^25^ showed that narigenin has antimicrobial activity against *H*. *seropedicae*. Naoumkina *el al*.^24^ reported that flavonone-3-β-hydroxylase was induced in rice following infection with *Xanthomonas oryzae*. In addition, nematode cysts stimulate isoflavone synthesis (including chalcone reductases, chalcone isomerase, isoflavon 2′-hydroxylase, isoflavones and isoflavone reductase synthase) in soybeans. Flavonols such as quercetin^23^ exhibit antimicrobial activity possibly by binding and inhibiting DNA gyrase^26^. The data thus indicate that down-regulation of flavonoid/isoflavone synthesis by *H*. *seropedicae* is part of the attenuation of the defence system in rice roots that is necessary to host an endophyte.

**Figure 3:**
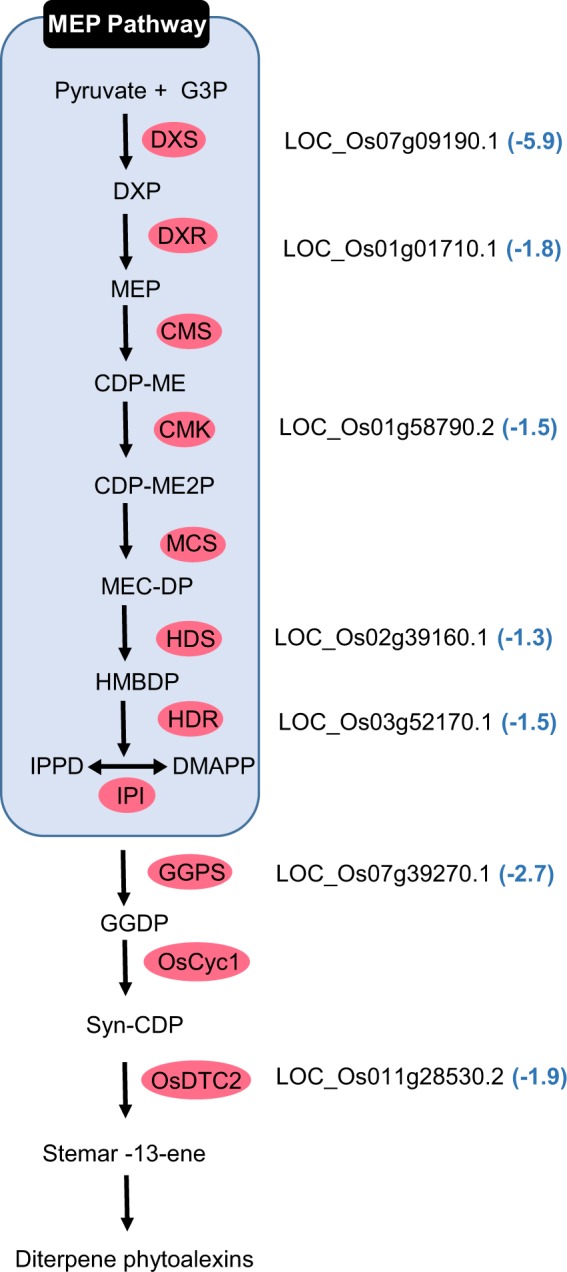
Isoprenoid synthesis genes down-regulated in rice roots colonised by *H*. *seropedicae*. The names of the genes differentially expressed are shown and the numbers in parentheses represent the fold change. The components of the MEP pathway leading to geranylgeranyl diphosphate synthesis and the diterpenoid- phytoalexin pathway are: G3P, glyceraldehyde-3-phosphate; DXP, 1-deoxy-D-xylulose 5-phosphate; MEP, 2-C-methyl-D-erythritol 4-phosphate; CDP-ME, 4-(cytidine 5-diphospho)-2-C-methyl-D-erythritol; CDP-ME2P, 2-phospho-4-(cytidine 5′-diphospho)-2-C-methyl-D-erythritol; MEC-DP, 2-C-methyl-D-erythritol 2,4-cyclodiphosphate; HMBDP, 1-hydroxy-2-methyl-2-butenyl 4-diphosphate; IPP, isopentenyl diphosphate; DMAPP, dimethylallyl diphosphate; GGDP, geranylgeranyl diphosphate; and CDP, copalyl diphosphate. Enzymes are indicated in rose coloured circles: DXS, 1-deoxy-D-xylulose 5-phosphate synthase; DXR, DXP reductoisomerase; CMS, CDP-ME synthase; CMK, CDP-ME kinase; MCS, MECDP synthase; HDS, HMBDP synthase; HDR, HMBDP reductase; IPI, IPP isomerase; GGPS, GGDP synthase; OsCyc1, syn-CDP synthase; OsCyc2, ent-CDP synthase; OsDTC2, stemar-13-ene synthase.

Isoprenoid (or terpenoid) synthesis was also modulated by *H*. *seropedicae* (Supplementary Table 4 and Fig. 3). Isoprenoids have diverse biological functions and are derived from isomeric compounds including isopentenyl diphosphate (IPP) and dimethylallyl diphosphate (DMAPP)^27^ via two pathways: the mevalonate pathway (MEV) and the methyl-erythritol phosphate pathway (MEP) (Fig. 3).

In rice, *H*. *seropedicae* repressed genes of the MEP pathway (Supplementary Table 4 and Fig. 3). The most repressed transcript (5.9 times) encodes 1-deoxy-D-xylulose 5-phosphate synthase (DXS) the first enzyme of the pathway that synthesises 1-deoxy-D-xylulose 5-phosphate (DXP) from pyruvate and D-glyceraldehyde 3-phosphate. DXP is a precursor of antimicrobial compounds including phytoalexins. Chitin induces the MEP pathway in cultured rice cells and as a result phytoalexins accumulate^28^. Furthermore, treatment of the cells with inhibitors of the enzymes DXS and 1-deoxy-D-xylulose 5-phosphate reductoisomerase (DXR) impeded chitin-dependent accumulation of phytoalexin. The authors suggested that activation of the MEP pathway is required to meet the demand for isoprenoid and phytoalexin synthesis in infected cells. In agreement with our results Drogue *et al*.^29^ showed the repression of a gene encoding stemar-13-ene synthase (OsDTC2), an enzyme involved in synthesis of phytoalexins in rice, which was repressed 1.9-fold in *H*. *seropedicae* treated rice roots (Fig. 3) and two-fold when infected with *Azospirillum* spp.^29^. It thus seems as if *H*. *seropedicae* represses production of defence-related isoprenoids in rice roots perhaps to allow the bacteria to enter and rapidly colonise the intercellular spaces and xylem.

#### Oxidative stress responses

In this study *H*. *seropedicae* modulated expression of five redox state genes including those for non-symbiotic hemoglobin 2 and peroxiredoxin. One peroxiredoxin transcript (LOC_Os07g44440.1) induced 3.7 and the other LOC_Os01g16152.1 was repressed 2.1 fold. Peroxiredoxins are a group of H_2_O_2_-decomposing antioxidant enzymes related to the redox state. In addition to the reduction of H_2_O_2_, peroxiredoxin proteins also detoxify alkyl hydroperoxides and peroxinitrite^30,31^.

Plants respond to attacks by pathogens with rapid increases in reactive oxygen species (ROS) such as superoxide and H_2_O_2_^32^. Peroxidases produce ROS that could cause oxidative damage to proteins, DNA, and lipids. Many defects in the immune system of mature *A*. *thaliana* plants with reduced expression of two key peroxidase genes, PRX33 or PRX34, were observed^33^. Silencing the French-bean class III peroxidase (FBP1) in *A*. *thaliana* impaired the oxidative burst and rendered plants more susceptible to bacterial and fungal pathogens^34^. Proteomic studies of rice roots seven days after inoculation with *H*. *seropedicae* showed induction of ascorbate peroxidases^35^, though the genes encoding these enzymes were not affected in our RNA-seq analyses. Moreover, seven days after inoculation, ROS levels in *Herbaspirillum rubrisubalbicans* attached to rice roots had increased suggesting that the bacteria were subject to oxidative stresses^36^. In this work, two peroxidase genes were induced by *H*. *seropedicae* in inoculated roots.

#### Cell wall

A variety of diazotrophic microorganisms such as *H*. *seropedicae* Z67, *H*. *rubrisubalbicans* and *A*. *brasilense* produce cell-wall degrading enzymes^6,18^. Interestingly, the *H*. *seropedicae* SmR1 genome did not reveal genes coding for known cellulases, pectinases or any other cell-wall degrading enzymes^37^. Nevertheless, rice roots inoculated with *H*. *seropedicae* induced a gene (2.0-fold) encoding a β-D-xylosidase and repressed 2.4-fold a gene coding for a polygalacturonase suggesting re-modeling of plant cell wall. In *A*. *thaliana*, β-D-xylosidase has been shown to be involved in secondary cell-wall hemi-cellulose metabolism and plant development^38^, but little is known about the function of this enzyme.

Other differentially expressed genes involved in cell-wall metabolism code for proteins similar to an expansin11 (induced 2.6-fold). Expansins have a loosening effect on plant cell-walls and function in cell enlargement as well as in diverse developmental processes in which cell-wall modification occurs including elongation. In addition, they promote elongation of root-hairs^39,40^ and root-hair initiation^41^.

Expansins have been correlated with plant-bacteria interactions. In tobacco, *Bacillus subtilis* G1, a plant growth promoting bacterium, induced the expression of two expansins NtEXP2 and NtEXP6^42^. Also, inoculation of *Melilotus alba* with *Sinorhizobium meliloti* lead to enhanced MaEXP1 mRNA levels in roots and nodules^43^. Together, these results suggest that inoculation with *H*. *seropedicae* also led to modification of plant cell wall which may facilitate bacterial colonization of inner tissue by loosening cell wall.

#### Plant immune responses

The plant immune system can sense and respond to pathogen attacks in two manners: the first involves recognition of pathogen/microbe associated molecular patterns (PAMPs) or damage associated molecular patterns (DAMPs) by surface pattern-recognition receptors (PRRs) resulting in pattern-triggered immunity (PTI). Second, resistance proteins (R) that recognise pathogen effectors are expressed leading to effector-triggered immunity (ETI)^44–47^. The receptors that perceive PAMPs or DAMPs have an ectodomain potentially involved in ligand binding, a single transmembrane domain and, most of times, a intracellular kinase domain^47^. Amongst the genes with the highest expression differences seen in RNA-seq data was a wall-associated receptor kinase-like (WAK) 22 precursor (LOC_Os10g07556.1), repressed 93-fold (6.5-fold by RT-qPCR) in *H*. *seropedicae* inoculated roots. Cell wall-associated receptor kinases (WAKs) contain an extracellular domain composed of one or more epidermal growth factor (EGF) repeats. Animal proteins containing these repeats are known to bind small peptides^48^. In *A*. *thaliana* WAKs bind to cross-linked pectin cell wall, pathogen- or damage-induced pectin fragments and oligogalacturonides, thus regulating cell expansion or stress response depending on the state of the pectin^49–51^. Several studies have described the role of WAK genes in rice resistance to pathogens^52–55^. Plant proteins domain architecture consist of cell-wall pectin binding extracellular region, the EGF-like domain, and a kinase domain. Analyses of rice loss-of-function mutants of WAK genes showed that individual genes are important for resistance against *M*. *oryzae*. OsWAK14, OsWAK91 and OsWAK92 positively regulate resistance while OsWAK112d is a negative regulator of blast resistance^55^. Cayrol *et al*. (2016) demonstrated that OsWAK14, OsWAK91 and OsWAK92 can form homo- and hetero-complexes and hypothesized that the loss of function of any of these proteins may destabilize the complex and affect their functioning. In *A*. *thaliana* the WAK22 orthologous gene (AtWAKL10) encodes a functional guanylyl cyclase which is co-expressed with pathogen defence related genes^56^. Thus, the wall-associated receptor kinase-like 22 gene of rice could be a candidate for surface pattern-recognition receptor and its repression may allow *H*. *seropedicae* to evade activation of the plant-defence system.

A cysteine-rich receptor-like protein kinase (CRK) (LOC_Os04g56430.1) and a serine/threonine-protein kinase At1g18390 precursor (LOC_Os05g47770.1) were induced 3.2-fold and 2-fold, respectively, while a lectin-like receptor kinase 7 (LOC_Os07g03790.1) and SHR5-receptor-like kinase (LOC_Os08g10320.1) were repressed 2.7 and 2.5-fold respectively in the presence of *H*. *seropedicae* (Supplementary Table 4). The latter protein has 75% identity with sugarcane SHR5 receptor kinase repressed by colonisation with diazotrophic endophytes^57^. The authors suggested that the expression levels of this gene were inversely related to the efficiency of beneficial plant-bacterial interactions^57^. Besides in *Arabidopsis* cysteine rich receptor-like kinase 5 protein is involved in regulation of growth, development, and acclimatory responses^58^.

Among the regulated genes that have functions involved in defence (in dark gray squares in Fig. 1B), as determined by MapMan, three genes encoding PR-proteins were repressed (LOC_Os10g25870.1, LOC_Os11g07680.1, LOC_Os02g38392.1, 2.5, 3.4 and 2.7-fold respectively) while one transcript that encoded a lipase called EDS1 (enhanced disease susceptibility 1) was induced 2-fold. The role of EDS1 in defence is well described in *A*. *thaliana* and is required for resistance conditioned by R genes which encode proteins that contain nucleotide-binding sites and leucine-rich repeats (NBS-LLR)^45,59–62^. Mutant *eds1* seedlings exhibited enhanced susceptibility to the biotrophic oomycete *Peronospora parasifica*^63^. Analysis of *EDS1* and *PR*-gene expression showed induction of both genes after inoculation with *Pseudomonas syringae* or treatment with salicylic acid (SA)^64^. In addition, in the *A*. *thaliana eds1* mutant, the expression of PR-proteins was undetectable. When the *eds1* mutant was treated with SA however, expression of PR was detected. The authors suggest that EDS1 functions upstream of *PR1*-mRNA accumulation. Among the 3 transcript for PR-proteins repressed, one (LOC_Os02g38392.1) is a NBS-LRR disease resistance protein. Therefore, in *H*. *seropedicae*- rice interaction an inverse correlation between PR-protein and EDS1 expression was observed, perhaps suggesting a fine regulation of these defence systems by *H*. *seropedicae*.

In previous work down-regulation of genes associated with defence was observed in rice plants inoculated with *H*. *seropedicae* such as a putative probenazole inducible protein (PBZ1, LOC_Os12g36840.1) that was repressed 3.6-fold as shown by RT-qPCR analysis^17^. The RNA-seq data showed that this transcript (LOC_Os12g36840.1) was repressed 2.9-fold. Another two transcript coding proteins similar to PBZ1 (LOC_Os12g36830.1 and LOC_Os12g36850.1) were also repressed (7.2 and 4.1, respectively). Kawahara *et al*.^65^ using an RNA-seq approach to study the transcriptome of rice inoculated with the blast fungus *M*. *oryzae* observed that the same PBZ1 genes detected in our study were induced 273 and 233-fold upon inoculation with the pathogen. The induction of PBZ1 gene by pathogens has been considered as a molecular marker for rice defence response^66^.

Another gene regulated by *H*. *seropedicae* that is related to defence codes for a thionin, a small cysteine-rich protein that occurs in a broad range of plant species^67^. Thionins are known for their toxicity to plant pathogens and several studies showed that their over-expression is related to increased resistance to diseases^68–71^. Brusamarello-Santos *et al*.^17^ showed 5-fold repression of thionin genes from chromosome 6 seven days after inoculation with *H*. *seropedicae* but these thionins from chromosome 6 were not regulated in roots three days after inoculation with *H*. *seropedicae*. Since there are 15 thionin genes (according to the Rice Genome Annotation Project RGAP7) sharing high identity in chromosome 6, the unmapped reads were mapped to the rice genome as well as to chromosome 6 separately. An average of 480 reads of control libraries and 136 of inoculated libraries mapped on thionin genes, a result that is in accordance with the repression pattern observed in rice roots seven days after inoculation with *H*. *seropedicae*^17^. Interestingly, a thionin transcript from chromosome 7 (LOC_Os07g24830.1) was induced 7.2-fold in the presence of *H*. *seropedicae* three DAI. Time-dependent regulation of thionin was also observed by Ji *et al*.^71^ in rice roots infected with *Meloidogyne graminicola*. Straub *et al*.^72^ observed only a few defence-related genes induced in the transcriptome of *Miscanthus sinensis* inoculated with *Herbaspirillum frisingense* helping to explain why this bacterium can effectively invade and colonise plants^72^.

The repression of genes related to defence is necessary perhaps to allow the bacteria to enter and rapidly colonise the intercellular space and xylem. However, it seems as if a balance between induction and repression of defence-related genes allows *H*. *seropedicae* to survive inside rice tissues while concomittantly activating some defence responses to protect the plant from pathogens.

#### Phytohormones

Auxins regulate diverse physiological processes such as vascular tissue differentiation, lateral root initiation and have also been linked to defence in plant-pathogen interactions^73–76^. Auxin response elements (AuxREs), when bound to auxin response factors (ARFs), control auxin-dependent gene expression. The Aux/IAA protein family members that inhibit ARFs mediate this regulation^77^.

Four differentially expressed transcript related to auxin signalling were identified in rice roots inoculated with *H*. *seropedicae*. The transcript LOC_Os08g24790.1 encoding an auxin-responsive protein was repressed 2.1-fold (Table 1) whereas the transcript coding for auxin-induced proteins, LOC_Os09g25770.1 and LOC_Os05g01570.1, were repressed 1.8 and 2.5-fold, respectively. Repression of genes related to auxin signalling was reported in rice roots inoculated with *H*. *seropedicae*^17^. Brusamarello-Santos *et al*.^17^ found that ARF2-like, IAA 11 and IAA18 were repressed 1.4, 1.5 and 2.8-fold, respectively, in the presence of *H*. *seropedicae* 7 days after inoculation. These genes were not regulated in our data probably due to the time difference of the cDNA library construction and expression levels too low.

**Table 1:** Rice genes modulated by colonisation with *H*. *seropedicae* that are involved in phyto-hormone signalling and the MTA cycle.

Locus name	Gene product/description*	Fold-change	Pvalue
LOC_Os01g67030.1	Auxin-responsive protein, putative, expressed	2.5	4.99E-06
LOC_Os08g24790.1	AIR12, putative, expressed	0.49	0.04
LOC_Os09g25770.1	Auxin-induced protein 5NG4, putative, expressed	0.55	0.01
LOC_Os05g01570.1	Auxin-induced protein 5NG4, putative, expressed	0.40	0.05
LOC_Os09g28050.1	1-aminocyclopropane-1-carboxylate synthase family protein (ACC synthase) (RAPDB) Asparate aminotransferase (MSU)	6.1	2.11E-54
LOC_Os03g63900.1	1-aminocyclopropane-1-carboxylate oxidase 2 (ACC oxidase)	0.50	7.88E-04
LOC_Os01g04800.1	B3 DNA binding domain containing protein (MSU) APETALA2/ethylene-responsive element binding protein 129 (RAPDB)	2.2	3.67E-03
LOC_Os06g02220.1	MTA/SAH nucleosidase	2.6	1.83E-11
LOC_Os04g57400.1	Methylthioribose kinase	6.1	3.20E-18
LOC_Os04g57410.1	Methylthioribose kinase	10.2	1.16E-12
LOC_Os06g04510.1	Similar to enolase 1	1.9	0.03
LOC_Os03g06620.1	1,2-dihydroxy-3-keto-5-methylthiopentene dioxygenase	7.5	5.94E-65
LOC_Os10g28360.1	1,2-dihydroxy-3-keto-5-methylthiopentene dioxygenase	0.47	6.14E-03
LOC_Os01g22010.3	S-adenosylmethionine synthetase (SAM)	1.9	1.72E-05

^*^Gene product name/description of the both rice database were used: MSU (Rice Genome Annotation Project) and RAPDB (The Rice Annotation Project).

*H*. *seropedicae* can produce IAA in the presence of tryptophan, thus suggesting the plant growth promotion effect may be due to bacterial derived auxin^78^. In addition exogenous application of auxin lead to increase in lateral root numbers^79^. In the absence of clear regulation of auxin-dependent genes and phenotype, the results suggest that auxin does not play an important role in Nipponbare rice roots colonization by *H*. *seropedicae*. On the other hand the repression of auxin signalling has been correlated to defence responses. Auxin levels have been correlated with susceptibility to pathogens^74^ that are able to produce high levels of auxin^80^. It has also been shown that pathogen-associated molecular patterns (PAMPs) induce the expression of a miRNA that negatively regulates mRNAs for F-box auxin receptors leading to resistance to *P*. *syringae* in *Arabidopsis*^81^. The observed repression in several genes involved with defence in the rice-*H*. *seropedicae* interaction opens the question whether auxin could be important for bacteria survival inside the plant by down-regulating defence system. Further studies are needed to elucidate if and how auxin signalling participates in plant-bacterial interactions.

Ethylene is also involved in several biological processes that activate defence responses and adventitious root-growth in rice and other plants^82–84^. Ethylene can be synthesised by oxidation of 1-aminocyclopropane-1-carboxylate (ACC) by ACC oxidase. ACC is synthesised from adenosylmethionine (AdoMet) by ACC synthase (ACS). ACS is up regulated 6-fold and ACC oxidase is repressed 2-fold by inoculation with *H*. *seropedicae*. An ethylene response factor (ERF) (LOC_Os01g04800.1) transcript was also induced 2.2-fold in inoculated roots. These data indicate that ethylene synthesis is attenuated in the presence of bacteria. Moreover Alberton *et al*.^35^ measured the level of ethylene in inoculated rice roots (seven days after inoculation) and found a decrease of ethylene levels. Furthermore, Valdameri *et al*.^36^ detected induction of ACC oxidase in rice plants inoculated with the pathogen *H*. *rubrisubalbicans*. These results suggest that the ethylene pathway is differentially modulated in the presence of pathogens and beneficial endophytic bacteria that promote plant growth.

Salicylic acid is derived from phenolic compounds and is involved in response to attack by pathogens^85,86^. In rice roots inoculated with *H*. *seropedicae*, a SA-dependent carboxyl methyltransferase family protein gene (LOC_Os11g15340.2) was repressed 40-fold. A member of this family is salicylic acid carboxyl methyltransferase (SAMT) that catalyses the formation of methyl salicylate (MeSA) from SA^87^. MeSA is an essential signal for systemic acquired resistance (SAR) in tobacco plants. In addition mutations in SAMT showed that this gene is required for SAR^88^.

SA signalling is differentially regulated by members of the WRKY transcription factor family^89^. In inoculated rice roots, two WRKY transcription factors were regulated, one repressed (2.3-fold) and one induced (2.0-fold). The induced gene encodes a WRKY51 similar to WRKY11 of *Arabidopsis*, whereas the repressed gene encodes WRKY46, which was shown to be induced by SA in *Arabidopsis*. WRKY11 is a negative regulator of resistance^90^ and *Arabidopsis* plants in which WRKY46 was over-expressed were more resistant to *P*. *syringae*^91^. These results are in agreement with the hypothesis that the SA signalling and defence system are attenuated in the presence of the *H*. *seropedicae*.

### Metal ion metabolism

**Figure 4:**
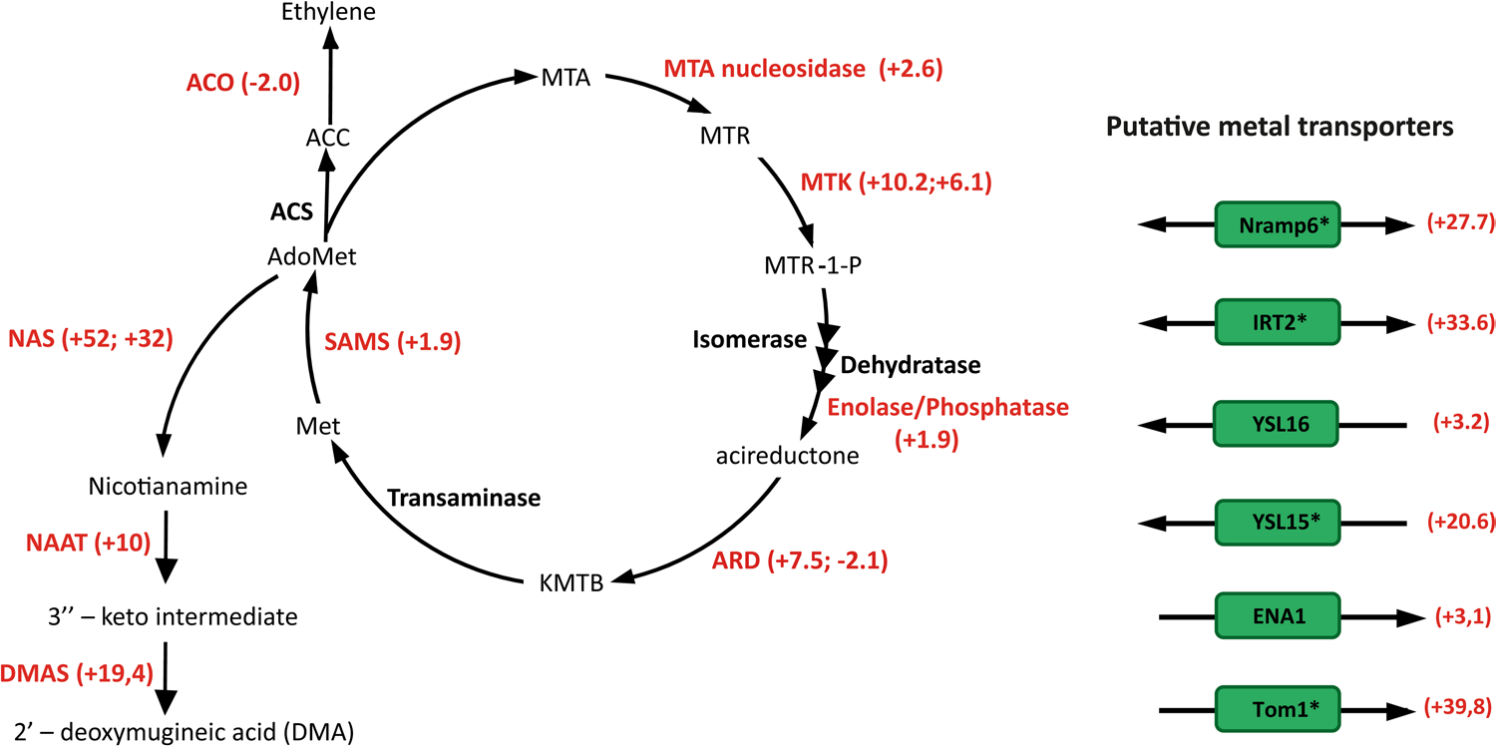
Differentially expressed genes in rice roots following colonisation by *H*. *seropedicae*. Genes involved in siderophore synthesis and transport, the methionine salvage pathway and ethylene synthesis are shown. Numbers in parentheses represent the fold change. *H*. *seropedicae* SmR1 induces methionine recycling and mugineic acid (MA) synthesis as well as the expression of transporters involved in iron metabolism. The expression of those genes marked with an asterisk was confirmed by RT-qPCR Abbreviations: AdoMet, S-adenosylmethionine; ACC, 1-aminocyclopropane-1-carboxylate; ACS, 1-aminocyclopropane-1-carboxylate synthase; ACO, 1-aminocyclopropane-1-carboxylate oxidase; MTA, 5′-methylthioadenosine; MTR, 5′-methylthioribose; MTK, methylthioribose kinase; MTR-1-P, 5′-methylthioribose-1-phosphate; KMTB, 2-keto-4-methylthiobutyrate; ARD, acireductone dioxygenase; SAMS, S-adenosylmethionine synthetase; NAS, nicotianamine synthase; NAAT nicotianamine aminotransferase; DMAS, deoxymugineic acid synthase; Tom1, transporter of mugineic acid 1; ENA1 (efflux transporters of nicotianamine 1); Nramp6, Natural Resistance-Associated Macrophage Protein; IRT2(iron-regulated transporter 2); YSL16 (yellow strip-like gene 16); YSL15 (yellow strip-like gene 15).

Several genes related to metal transport were differentially expressed, most of them up regulated in the presence of *H*. *seropedicae*. Amongst the 20 most highly regulated rice genes, eight were related to metal transport (Table 2 and 3). Phyto-siderophore synthesis starts with production of nicotianamine (NA) from S-adenosylmethionine, which in turn is derived from 5′-methylthioadenosine of the methionine salvage pathway (MTA cycle) (Fig. 4). Transcripts of enzymes of the MTA cycle encoding SAM, MTA nucleosidase, MTR kinase, E1 enolase/phosphatase and acireductone dioxygenase (ARD) were induced 1.9, 2.6, 6.0, 1.9 and 7.5-times, respectively, in roots colonised by *H*. *seropedicae* (Fig. 4). Using proteomic and RT-qPCR analyses, Alberton *et al*.^35^ observed similar expression patterns in rice roots inoculated with *H*. *seropedicae*.

**Table 2:** Rice genes highly regulated (fold change > 10) by inoculation with *H*. *seropedicae*.

Locus name	Gene product/description*	Fold change	P-value
**Up-regulated rice genes**			
LOC_Os02g20360.1	Ttyrosine aminotransferase, putative, expressed, Similar to Nicotianamine aminotransferase A (RAPDB)	10	5.24E-30
LOC_Os04g57410.1	Methylthioribose kinase, putative. Expressed	10.2	1.16E-12
LOC_Os03g26210.1	Helix-loop-helix DNA-binding domain containing protein. Expressed	12.6	4.10E-09
LOC_Os01g72370.1	Helix-loop-helix DNA-binding domain containing protein. Expressed	16.4	3.36E-74
LOC_Os11g15624.1	Expressed protein	17.7	1.22E-25
LOC_Os03g13390.2	Oxidoreductase. aldo/keto reductase family protein, putative. Expressed	19.4	1.61E-10
LOC_Os02g43410.1	Transposon protein, putative. Unclassified. Expressed	21	3.97E-81
LOC_Os10g11889.2	Expressed protein	23	3.29E-113
LOC_Os01g65110.1	POT family protein. Expressed	26	6.63E-14
LOC_Os07g15460.1	Metal transporter Nramp6, putative. Expressed	27	3.56E-61
LOC_Os03g03724.1	Expressed protein	28	0.02
LOC_Os03g19427.1	Nicotianamine synthase, putative. expressed	32	1.02E-22
LOC_Os03g46454.1	Metal cation transporter, putative. expressed	34	4.02E-08
LOC_Os06g19095.1	Expressed protein	40	1.51E-14
LOC_Os11g04020.1	Major facilitator superfamily antiporter, putative. expressed	40	8.84E-51
LOC_Os03g19420.2	Nicotianamine synthase, putative. expressed	52	5.57E-80
LOC_Os12g18410.1	Expressed protein	70	2.07E-05
LOC_Os03g12510.1	Non-symbiotic hemoglobin 2, putative. expressed	104	5.58E-24
LOC_Os04g51660.1	Transferase family protein, putative, expressed	0.08	2.79E-03
LOC_Os04g59020.1	Integral membrane protein, putative, expressed	0.08	3.41E-03
LOC_Os09g23300.1	Integral membrane protein, putative, expressed	0.06	3.63E-05
LOC_Os11g15340.2	SAM dependent carboxyl methyltransferase family protein, putative, expressed	0.03	1.54E-03
LOC_Os01g55690.1	Glutelin, putative, expressed	0.02	0.02
LOC_Os12g38040.1	Metallothionein family protein, expressed	0.02	3.72E-22
LOC_Os10g07556.1	Wall-associated receptor kinase-like 22 precursor, putative, expressed	0.01	5.22E-08

^*^Gene product name/description of the both rice database were used: MSU (Rice Genome Annotation Project) and RAPDB (The Rice Annotation Project).

**Table 3:** Differentially expressed genes in rice roots colonised by *H*. *seropedicae* involved in uptake and transport of metals.

Locus name	Gene product/description*	Fold change	p-value
LOC_Os01g22010.3	S-adenosylmethionine synthetase, putative, expressed	1.9	1.72E-05
LOC_Os03g19427.1	Nicotianamine synthase. putative. expressed	32	1.02E-22
LOC_Os03g19420.2	Nicotianamine synthase. putative. expressed	52	5.57E-80
LOC_Os02g20360.1	tyrosine aminotransferase, putative, expressed (MSU), Similar to Nicotianamine aminotransferase (RAPDB)	10	5.24E-30
LOC_Os03g13390.2	Oxidoreductase. aldo/keto reductase family protein, putative. Expressed (MSU) Similar to NADPH-dependent codeinone reductase, gene name: deoxymugineic acid synthase1 (RAPDB)	19.4	1.61E-10
LOC_Os11g04020.1	Major facilitator superfamily antiporter, putative, expressed (TOM1)	40	8.84E-51
LOC_Os11g05390.1	Transporter, major facilitator family, putative, expressed (ENA1)	3.1	5.80E-03
LOC_Os03g46470.1	Metal cation transporter, putative, expressed (OsIRT1)	4.3	4.09E-14
LOC_Os03g46454.1	Metal cation transporter, putative, expressed (OsIRT2)	34	4,02E-08
LOC_Os04g45900.1	Transposon protein, putative, unclassified, expressed (MSU) Similar to Metal-nicotianamine transporter YSL2, Gene symbol synonym: OsYSL16 (RAPDB)	3.2	1.23E-07
LOC_Os02g43410.1	Transposon protein, putative. Unclassified. Expressed (MSU) Iron-phytosiderophore transporter, Iron homeostasis (Os02t0650300-01); Similar to Iron-phytosiderophore transporter YSL15. (Os02t0650300-02) (RAP-DB)	21	3.97E-81
LOC_Os07g15460.1	Metal transporter Nramp6, putative. Expressed	27	3.56E-61

^*^Gene product name/description of the both rice database were used: MSU (Rice Genome Annotation Project) and RAPDB (The Rice Annotation Project).

The synthesis of NA and MAs involves a set of enzymes including S-adenosylmethionine synthase (SAM) that catalyses the adenylation of L-methionine to S-adenosylmethionine, nicotianamine synthase (NAS) that converts S-adenosylmethionine to nicotianamine, and nicotianamine aminotransferase (NAAT) that catalyses the amino transfer of NA to produce the 3”-keto intermediate that is reduced by deoxymugineic acid synthase (DMAS) to produce 2′-deoxymugineic acid (DMA)^92–95^.

Two nicotianamine synthase (NAS) genes of rice - LOC_Os03g19420.2 and LOC_Os03g19427.1 were induced (52- and 32-fold, respectively) in inoculated rice roots. LOC_Os03g19427.1 was also induced in rice roots inoculated with *Azospirillum spp*.^29^. Increased levels of nicotianamine have been shown to increase Fe uptake in rice plants^96^. Furthermore, in *Lotus japonicus* inoculated with *Mesorhizobium loti*, nicotianamine synthase 2 was expressed only in nodules pointing to a role in symbiotic nitrogen fixation^97^. A tomato mutant defective in the synthesis of nicotianamine was affected in iron metabolism^98^. In addition, NAAT (LOC_Os02g20360.1) and DMAS (LOC_Os03g13390.2) were also induced 10-fold and 19-times respectively in colonised rice roots. Iron deficiency provoked induction of rice gene OsNAAT1^95^.

Recently, members of a major facilitator super-family have been described as essential to the efflux of MA and NA in rice. TOM1 (transporter of mugineic acid 1) is involved in the efflux of MA, while ENA1 (efflux transporters of nicotianamine 1) and ENA2 in the efflux of NA^99^. TOM1 (LOC_Os11g04020.1) and ENA1 (LOC_Os11g05390.1) were induced 40 and 3-fold in colonised roots. Induction of TOM1 was confirmed by RT-qPCR (Fig. 2).

Fe^+++^ ions chelated by PS need to be transported inside the cell. In gramineous plants, two groups of Fe-MA transporters are present: ZmYS1^100^ and the YSL (yellow strip-like) transporter family^101^. Inoue *et al*.^101^ analysed the expression of 18 YSL genes in rice and observed induction of OsYSL15 and OSYSL16 genes under iron-deficiency. Other studies have shown that OsYSL2 (LOC_Os02g43370) takes up Fe^2+^-NA^102^ and OsYSL16 Fe^3+^-DMA (LOC_Os04g45900.1)^103^. Here we showed induction (3.2-fold) of OsYSL16 (LOC_Os04g45900.1) and 21-fold increase of transcripts encoding a gene similar to OSYSL15 (LOC_Os02g43410.1). Induction of OSYSL15 was confirmed by RT-qPCR (Fig. 2). Furthermore, OsIRT1 (iron-regulated transporter 1) and OsIRT2 were also induced under low Fe conditions^104^. We detected induction of OsIRT2 (LOC_Os03g46454.1) (34-fold in RNA-seq and 93-fold in RT-qPCR) (Fig. 2).

Other metal transporters such as Nramp6 (LOC_Os07g15460.1) were also up regulated (27-fold) in colonised rice roots, a result confirmed by RT-qPCR (Fig. 2). Members of the natural resistance-associated macrophage protein (NRAMP) family are transition metal cation/proton co-transporters or anti-porters of broad specificity. AtNRAMP6 of *Arabidopsis* is up-regulated in response to iron deficiency and is involved with metal mobilization from vacuoles to cytosol^105^. Induction of Nramp6 was observed in rice roots colonised by *H*. *seropedicae*. In a previous study in rice inoculated with *Azospirillum* spp the expression of this gene was also induced^29^. In addition a gene for an integral membrane protein (LOC_Os09g23300.1) named OsVIT2 was 17.7-fold repressed by *H*. *seropedicae*. This gene is involved in transport of Fe/Zn into vacuoles and is up-regulated in rice roots with excess Fe. Knockout/ knockdown of this gene led to Fe accumulation in seeds^106,107^. Together, these results suggest that colonised roots respond in such a manner as to accumulate Fe.

Interestingly the transcript with the highest fold change [104-times – a result confirmed using RT-qPCR (Fig. 2)] in colonised roots codes for the non-symbiotic hemoglobin 2 (LOC_Os03g12510.1). High levels of non-symbiotic hemoglobin 2 could help buffer free oxygen and protect bacterial nitrogenase. Arredondo-Peter *et al*.^108^ studied the repression of two hemoglobins, Hb1 and Hb2, in rice. The Hb2 induced by *H*. *seropedicae* is very similar to the one described by Arredondo-Peter *et al*. (1997) (coverage 82% with an identity of 97%). In addition, Lira-Ruan, Sarath and Arredondo-Peter^109^ studied the synthesis of hemoglobins in rice under normal and stress conditions, coming to the conclusion that Hbs are not part of a generalised stress response. They demonstrated that Hb1 is expressed in different rice organs (root and leaves) during plant development. In etiolated rice plants under 0_2_ limiting conditions the Hb levels increase^109^. This increase suggest that Hb expression maybe due to reduced O_2_ levels in the presence of the bacteria which make the root environment microaerophilic. The higher requirement for Fe needed for incorporation into Hb may partially explain activation of siderophore synthesis and Fe accumulation. We also found a *H*. *seropedicae* bacterioferritin (Hsero_1195) gene induced (2.2-fold), but symptoms of Fe deficiency in colonised rice plants were not observed.

Iron homeostasis has been related to plant defence, ROS accumulation and immunity. Also, Fe deficiency triggers accumulation of antimicrobial phenolics compounds. It has been suggested recently that Fe sequestration by bacterial siderophore could be a signal for pathogen infection^110^. However, bacterial genes involved in siderophore biosynthesis were not observed among the *H*. *seropedicae* genes expressed in rice roots. Also, transcriptomic analysis of *H*. *seropedicae* attached to wheat and mayze roots did not show iron metabolism genes up-regulated^111,112^. These results suggest that the effect of bacteria on plant iron mebabolism is more complex than those caused by iron sequestration.

### *H*. *seropedicae* transcripts detected in rice roots

The libraries from inoculated roots were mapped against the *H*. *seropedicae* genome (24,263 reads representing 0.06% of the total reads) (Supplementary Table 1). Amongst the 4,085 annotated genes of *H*. *seropedicae*, 287 were expressed in rice roots (at least one-fold coverage (this set of genes was called *H*. *seropedicae* expressed genes) (Supplementary Table 5). After ribosomal genes, the most abundant functional classes found were unknown, energy production and conversion, amino acid transport and metabolism, cell motility and cell wall (Table 4). Comparison of expressed genes of *H*. *seropedicae* detected in plants with bacterial genes expressed in culture revealed only 16 differences [p-value < 0.05 using the DESeq statistical package (Table 4)].

**Table 4:** Genes of *H*. *seropedicae* regulated during interaction with rice roots.

Locus_tag	Fold Change	p-value	ID Feature	Description	COG
HSERO_RS17470	0.4	7.0E-04	*qor*	qor NADPH:quinone oxidoreductase protein 4007887:4008915 forward	C;R - Energy production and conversion;General function prediction only
HSERO_RS05670	30	6.20E-03	Hsero_1130	Hsero_1130 ABC-type dipeptide transporter, periplasmic peptide-binding protein 1287409:1289031 forward	E - Amino acid transport and metabolism
HSERO_RS23580	27	7.70E-03	*urtA*	UrtA ABC-type urea transport system, periplasmic component protein 5407999:5409252 forward	E - Amino acid transport and metabolism
HSERO_RS00420	43	0.02	*glnK*	GlnK nitrogen regulatory PII-like protein 99052:99390 forward	E - Amino acid transport and metabolism
HSERO_RS07390	0.2	2.0E-04	*ttuC*	TtuC tartrate dehydrogenase protein 1690575:1691654 reverse	G - Carbohydrate transport and metabolism
HSERO_RS05635	84	0.02	Hsero_1123	Hsero_1123 family II aminotransferase protein 1278563:1279939 reverse	H - Coenzyme transport and metabolism
HSERO_RS07590	0.1	2.0E-03	*rplS*	RplS 50S ribosomal subunit protein L19 1731532:1731915 forward	J - Translation, ribosomal structure and biogenesis
HSERO_RS03380	0.3	0.02	*ompW2*	OmpW2 outer membrane W protein 741190:741927 forward	M - Cell wall
HSERO_RS12905	0.4	5.0E-03	*lon*	Lon ATP-dependent protease LA protein 2940893:2943301 reverse	O - Posttranslational modification, protein turnover, chaperones
HSERO_RS00425	20	0.04	*amtB*	AmtB ammonium transporter transmembrane protein 99406:100938 forward	P - Inorganic ion transport and metabolism
HSERO_RS14575	0.1	1.10E-03	Hsero_2905	Hsero_2905 conserved hypothetical protein 3301039:3301272 reverse	S - Function unknown
HSERO_RS00415	15	0.03	Hsero_0083	Hsero_0083 membrane protein 98251:99039 forward	S - Function unknown
HSERO_RS13665	90	1.90E-03	Hsero_2723	Hsero_2723 methyl-accepting chemotaxis transmembrane protein 3101951:3103597 reverse	T;N - Signal transduction mechanisms;Cell motility
HSERO_RS06095	0.3	0.04	*trnK*	TrnK tRNA-Lys 1374570:1374645 reverse	trnK
HSERO_RS07375	0.2	1.7E-03	*trnL*	TrnL tRNA-Leu 1689660:1689744 reverse	trnL
HSERO_RS07775	0.1	0.03	*trnS*	TrnS tRNA-Ser 1781783:1781873 forward	trnS

Genes involved in nitrogen fixation (*nif*) were found amongst genes classified as “energy production and conversion” and “amino acid transport and metabolism”. *Nif*-genes encode proteins involved in the synthesis, maturation and assembly of the nitrogenase complex^3^. *nifD* and *nifH* (coverage 1.7 and 1.2 respectively) were highly expressed in *H*. *seropedicae* colonising wheat and maize roots^111,112^. Two ferredoxin genes (*fdxA* and *fdxN*, coverage 1.9 and 1.6 respectively) important for nitrogenase activity were also identified^113^. The promoters of *nif* genes are activated by NifA that, in turn, is regulated by the Ntr system. When comparisons were made between free-living *H*. *seropedicae* and those grown in association with rice, *glnK* and *amtB* of the Ntr system were induced 43 and 20-times respectively in the plant-bacterial interaction. Pankievicz *et al*.^111^ also found that *amtB* was induced in *H*. *seropedicae* attached to maize roots. Furthermore, *fixN* and *fixP* (both 1.6X coverage) were also detected *in planta* along with an *urtA* ABC-type urea transport system (Hsero_4713) (27-fold of induction *in planta*).

Twenty-three genes related to cell motility were found, four with >2X coverage. Amongst these were *cheW* (a positive regulator of CheA), *flhD*, *fliC* and *pilZ* (Hsero_2062) that encodes a type IV *pilus* assembly protein. A methyl-accepting chemotaxis trans-membrane protein (Hsero_2723) was induced 90-fold when compared with expression in culture^114^. This gene was also found to be up-regulated in epiphytic *H*. *seropedicae* colonising wheat and maize^111,112^. Methyl-accepting chemotaxis proteins interact with Che proteins to detect signals from the environment.

Among the 19 cell-wall related genes, seven were covered at least 2-fold and an outer-membrane porin (Hsero_4295) was induced 28-fold. Another membrane-protein (Hsero_0083) of unknown function was induced 14-fold. These could be proteins that *H*. *seropedicae* uses to recognise rice.

## Materials and Methods

### Plant material and growth conditions

Testas were removed from seeds of rice (*Oryza sativa* ssp *japonica* cv. Nipponbare, kindly provided by the Instituto Riograndense do arroz, IRGA – Avenida Missões 342, Porto Alegre, RS, Brazil), then disinfected with 70% (v/v) ethanol for 5 min followed by 30 min soaking in 8% sodium hypochlorite (1 mL per seed) containing 0.1% v/v Triton-X100. After rinsing 20 times with sterile water, the seeds were treated with 0.025% (v/v) Vitavax-Thiram (Chentura, Avenida Nações Unidas 4777, Alto de Pinheiros, São Paulo, SP, Brazil) fungicide solution and stirred (120 rpm) for 24 h in the dark at 30 °C. The seeds were then transferred to 0.7% water-agar and left for two days to germinate after which the seedlings were inoculated with 1 mL of *Herbaspirillum seropedicae* strain SmR1 (10^8^ cells/seedling) for 30 minutes while control seedlings were treated with 1 mL of N-free NFbHP-malate medium^115^ (controls). Seedlings were washed with sterile water and transferred to glass tubes (25 cm long, 2.5 cm diameter) containing propylene beads and 25 mL of modified Hoagland’s solution^116^ without nitrogen (1 mM KH_2_PO_4_, 1 mM K_2_HPO_4_, 2 mM MgSO_4_.7H_2_O, 2 mM CaCl_2_.2H_2_O, 1 mL/L micronutrient solution (H_3_BO_3_ 2.86 g.L^−1^, MnCl_2_.4H2O 1.81 g.L^−1^, ZnSO_4_.7H_2_O 0.22 g.L^−1^, CuSO_4_.5H_2_O 0.08 g.L^−1^, Na_2_MoO_4_.2H_2_O 0.02 g.L^−1^) and 1 mL.L^−1^ Fe-EDTA solution (Na_2_H_2_EDTA._2_H_2_O 13.4 g.L^−1^ and FeCl_3_.6H_2_O 6 g.L^−1^)), pH 6.5–7.0. Plants were cultivated at 24 °C under 14 h light and 10 h dark for 3 days. *H*. *seropedicae* was cultivated in NFbHP malate medium containing 5 mM glutamate as the nitrogen source. Cells were shaken (120 rpm) overnight at 30 °C, then centrifuged, washed once with N-free NFbHP-malate and suspended in the same medium to OD_600_ = 1 (corresponding to 10^8^ cells.mL^−1^). Strain SmR1^117^ is a spontaneous streptomycin resistant mutant of strain Z78^118^. For colonisation assays roots from one plant were washed in 70% v/v ethanol for 1 min, 1% chloramine T for 1 min followed by three washes with sterile water^119^. At least 5 plants were used per data point. The roots were then crushed with a mortar and pestle in 1 mL of NFbHP-malate and serial dilutions (10^−1^ to 10^−5^) were plated onto solid NFbHP-malate containing 20 mM NH_4_Cl. Two days later the cells were counted to determine the colony forming units (CFU) per gram of fresh root.

### RNA isolation and construction of libraries

Three days after inoculation with *H*. *seropedicae* (6 days after the disinfection procedure) the roots were separated from the aerial part and immediately stored in RNA later™ (Life Technologies, Foster City, CA, USA). Total RNA was extracted from roots of five rice plants for each biological replicates (using a RNAqueous kit (Ambion, Austin, TX, USA). Contaminating genomic DNA was eliminated with RNase-free DNase I (Ambion) for 30 min at 37 °C. Total RNA (7 to 10 µg) was depleted of ribosomal RNA by treatment with a RiboMinus™ Plant Kit for RNA-Seq (Invitrogen, Carlsbad, CA, USA). The integrity and quality of the total RNA was checked spectrophotometrically and by agarose gel electrophoresis. Whole Transcriptome Analysis RNA Kits™ (Life Technologies) were used on 500 ng purified RNA to construct the libraries and sequencing was performed in a SOLiD4 (Life Technologies) sequencer. Two independent libraries were constructed for each condition (control and inoculated). Three of these libraries (two from control sample and one from inoculated sample) were run twice to check for technical reproducibility.

### Sequencing and data analysis

SOLiD sequencing produced 161 million 50 bp reads that were analysed by SAET software (Applied Biosystems – Foster City, CA, EUA) to improve base calling (command shown in Supplementary material 1), followed by quality trimming using the CLC Genomics Workbench (CLC bio, a QIAGEN Company, Silkeborgvej 2, DK-8000 Aarhus C, Denmark) (quality scores higher than 0.05 and reads with less than 20 bp were discarded). Then the reads were mapped to the rice genome database from the MSU Rice Genome Annotation Project (http://rice.plantbiology.msu.edu/) using CLC Genomics Workbench and the following parameters: a minimum length fraction of 95%, minimum similarity of 90% and only one hit. Differential expression was analysed using DESeq.^19^, genes covered at least twice were considered expressed and regulated when expression changed two times and the p-value was lower than 0.05. For Blast2GO analyses, protein sequences encoded by differentially expressed genes were used to search non-redundant (nr) database with BLASTp. Then, Gene Ontology (GO) terms were assigned based on the top BLAST hit using Blast2GO^20^ with default parameters. The results of GO annotation were combined for Gene Set enrichment analysis (GSEA)^21^ using Blast2GO (default parameters), which identifies GO terms enriched as a result of differential expression between samples. A heat map showing the expression profiles of the differentially expressed genes normalized by CPM (counts per million) was constructed using an in-house script (Supplementary material 2). MapMan analyses were performed with differentially expressed transcript table as input using default parameters.

### Quantification of mRNA levels using RT-qPCR

Reverse transcription quantitative PCR (RT-qPCR) analyses were used to evaluate gene expression under the conditions described above. Total RNA was isolated from rice roots using the TRI Reagent (Sigma, St. Louis, MO, USA) and contamination with genomic DNA was removed with DNase I (Life Technologies). The integrity and quality of the total RNA was confirmed by spectrophotometric analyses and electrophoresis. cDNA was produced from 1 μg DNase-treated total RNA using high-capacity cDNA reverse transcription kits (Life Technologies). The cDNA reaction was diluted 60 times before quantitative PCR using Power SYBR-Green PCR Master Mix on a Step One Plus Real Time-PCR System (both from Life Technologies). Primer sequences are listed in Supplementary Table 6 and were designed with the Primer express 3.0 software (Applied Biosystems) and the NCBI primer designing tool using the genome sequence of *O*. *sativa* ssp. japonica cv. Nipponbare. Calibration curves for all primer sets were linear over four orders of magnitude (R^2^ = 0.98 to 0.99) and efficiencies were 90% or higher. mRNA expression levels were normalised using the expression levels of actin 1, tubulin beta-2 chain (beta-2 tubulin)^120^ and a hypothetical protein (protein kinase)^121^ using geNorm 3.4 software^122^. The relative expression level was calculated according to Pfaffl^123^. Three independent samples were analysed for each condition and each sample was assayed in triplicate.

## Supplementary information

Supplementary information

Dataset 1

Dataset 2

Dataset 3

## Data Availability

The data that support the findings of this study are openly available. RNA-Seq data from this study have been deposited at the NCBI under the BioProject accession No. PRJNA489273 and BioSample Nos. SAMN09942067, SAMN09942069, SAMN09942071 and SAMN09953899.
