# Supplementary material for: Modulation of defence and iron homeostasis genes in rice roots by the diazotrophic endophyte Herbaspirillum seropedicae
Source: Sci Rep. 2019 Jul 22;9:10573. doi: 10.1038/s41598-019-45866-w (PMC6646362; doi:10.1038/s41598-019-45866-w)
Supplement: Supplementary file 1 — Supplementary information [file 41598_2019_45866_MOESM1_ESM.pdf]

Supplementary information

Modulation of defence and iron homeostasis genes in rice roots by the diazotrophic endophyte *Herbaspirillum seropedicae*

Liziane Cristina Campos Brusamarello-Santos[1,3], Dayane Alberton[2], Glaucio Valdameri[2], Doumit Camilios-Neto[1,6], Rafael Covre[3], Katia de Paiva Lopes[3,7], Michelle Zibetti Tadra-Sfeir[1], Helisson Faoro[1,8], Rose Adele Monteiro[1], Adriano Barbosa-Silva[3,9], William John Broughton[4], Fabio Oliveira Pedrosa[1], Roseli Wassem[5], Emanuel Maltempi de Souza[1]*

[1]Department of Biochemistry and Molecular Biology, Federal University of Parana, Curitiba, PR, Brazil

[2]Department of Clinical Analysis, Federal University of Parana, Curitiba, PR, Brazil

[3]Sector of Professional and Technological Education, Federal University of Parana, Curitiba, PR, Brazil

[4]Federal Institute of Materials Research and Testing, Division 4 Environment, Berlin, Germany

[5]Department of Genetic, Federal University of Parana, Curitiba, PR, Brazil

[6]Present address: Department of Biochemistry and Biotechnology, State University of Londrina, Londrina, PR, Brazil

[7]Present address: Department of Neuroscience, Icahn School of Medicine at Mount Sinai, New York, United States

[8]Present address: Carlos Chagas Institut – Fiocruz, Curitiba, PR, Brasil

[9]Present address: Luxembourg Centre for Systems Biomedicine (LCSB), University of Luxembourg, Esch-sur-Alzette, Luxembourg.

* Correspondence and requests for materials should be addressed to Emanuel Maltempi de Souza (E-mail address: souzaem@ufpr.br)

Supplementary material

Supplementary material 1: Command line used for SAET software

```
$ /home/liziane/saet_mp/ home/liziane/cr3a.csfasta /home/liziane/cr3a.qual 382788128 -fixdir /home/liziane/cr3a_fixdir
```

Supplementary material 2: Script used for heat map construction.

```
# Heatmaps for rice dataset
library(edgeR)
require(gplots)
require(Hmisc)
library(RColorBrewer)
library(ComplexHeatmap)

setwd("/Users/Liziane/Documents/2019/revisão-artigo-arroz/heatmap/katia")
counts_table = read.table("input_DESeq_3dias_cob2.txt", header = T, row.names = 1)
dim(counts_table) #13840     7
deg_genes = read.table("LOCs_rice.txt", header = T)
dim(deg_genes) #1014 Differentially Expressed genes

###Remove genes with no expression
counts_table_nozero = counts_table[rowSums(counts_table)!=0,]
dim(counts_table_nozero) #13840     7
cpm_rice = cpm(counts_table_nozero)

###Heatmaps###
#Spearman
cormatrix = rcorr(as.matrix(cpm_rice), type='spearman')
corrdata = as.matrix(cormatrix$r)

pdf(file = "CPM_Spearman.pdf",width = 16,height = 12, fonts = "sans",pointsize = 16)
heatmap.2(corrdata, main="CPM - SPEARMAN",trace="none", col = c(sort(brewer.pal(9,"Blues")),brewer.pal(9,"Reds")), margins = c(15,15))
```

```
dev.off()

#Pearson
cormatrix = rcorr(as.matrix(cpm_rice), type='pearson')
corrdata = as.matrix(cormatrix$r)

pdf(file = "CPM_Pearson.pdf",width = 16,height = 12, fonts = "sans",pointsize = 16)
heatmap.2(corrdata, main="CPM - PEARSON",trace="none", col =
c(sort(brewer.pal(9,"Blues")),brewer.pal(9,"Reds")), margins = c(15,15))
dev.off()

#By DE gene list
deg_genes_exp = cpm_rice[which(rownames(cpm_rice)%in%deg_genes$genes),]

pdf(file = "CPM_genesDE.pdf",width = 6,height = 8, fonts = "sans",pointsize = 16)
Heatmap(log10(deg_genes_exp+1),
        show_row_names = FALSE,
        name = "log10(CPM+1)",
        col = c(sort(brewer.pal(9,"Blues")),brewer.pal(9,"Reds")))
dev.off()

png(("CPM_genesDE.png"),width = 6,height = 8, res = 300, units = "in")
Heatmap(log10(deg_genes_exp+1),
        show_row_names = FALSE,
        name = "log10(CPM+1)",
        col = c(sort(brewer.pal(9,"Blues")),brewer.pal(9,"Reds")))
dev.off()
```

Supplementary Figure

Supplementary Fig.1 Colony-forming units of *H. seropedicae* colonizing rice roots epiphytically (red line) and endophytically (blue line) 1, 3 and 7 days after inoculation (DAI). The results are average of at least 5 independent replicates.

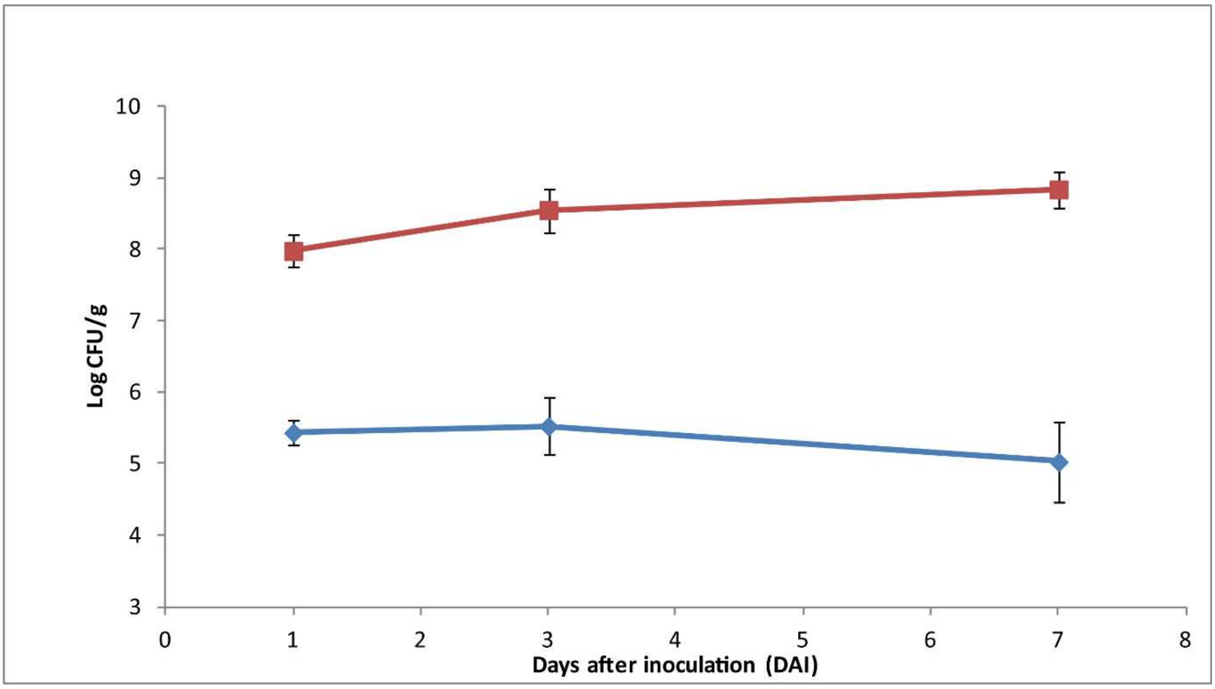

Supplementary Fig.2. Diagram showing how rice roots were grown, inoculated with *H. seropedicae*, prepared for extraction of RNA and the RNA-seq analyses performed.

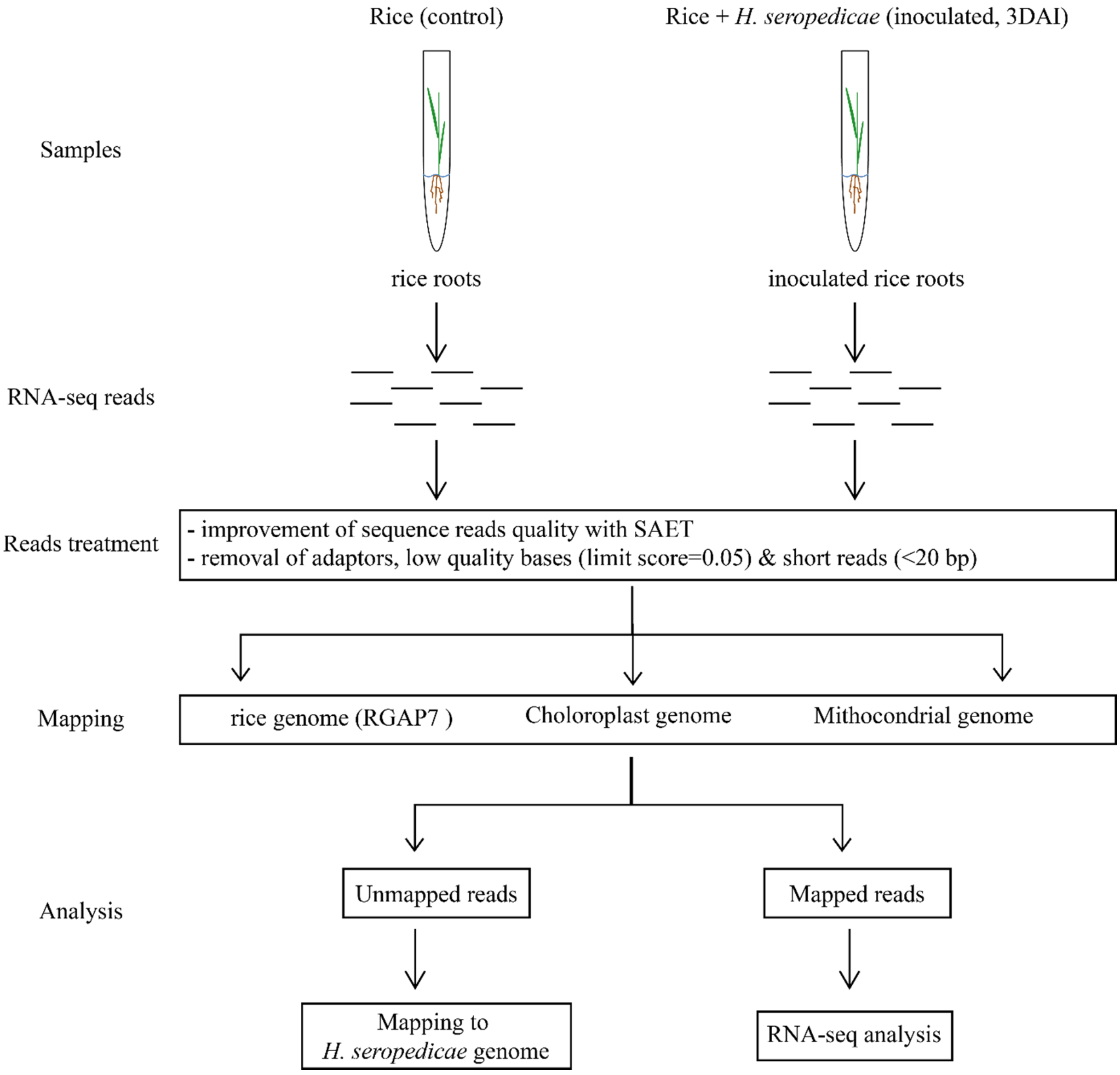

Supplementary Fig.3. Heat map showing the expression profiles of rice roots colonised by H. seropedicae and controls. Only the differentially expressed genes (p-value <0.05, fold change > 2, n=255) were used and the data were normalized by CPM (counts per million). Dark blue colors represents lower expression, and dark-red colour corresponds to high expression, in a log10 scale. CR3a and CR3b are independently biological replicate libraries from non-colonised roots (controls) and and IR3a and IR3b are biological replicate libraries from colonised roots. Run 1 and Run 2 are technical sequencing runs of the libraries.

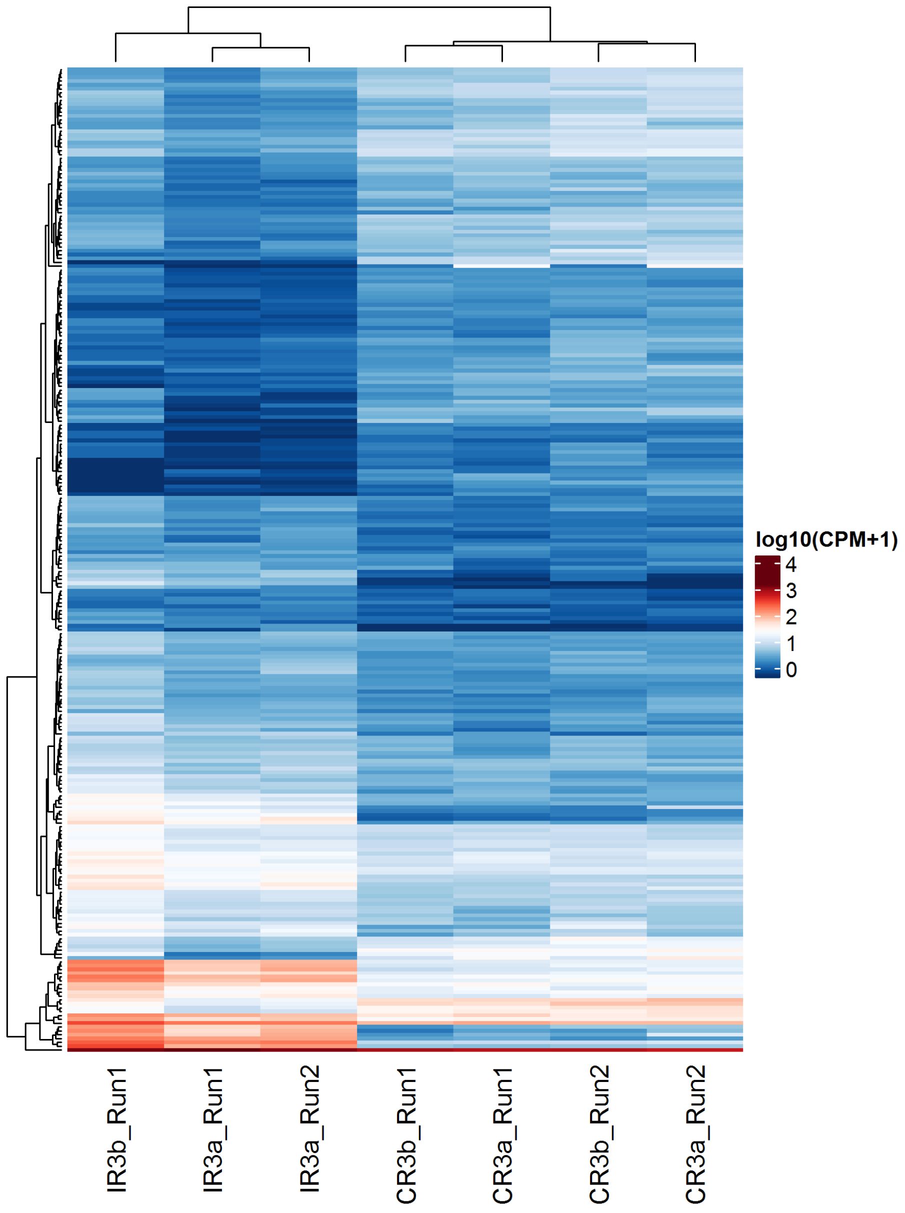

Supplementary Tables

Supplementary Table 1 – Statistics of RNA-seq reads mapping

	Control	Inoculated
**Total number of reads**	101,587,721	59,591,404
**Total number of reads after trimming**	65,099,320	38,463,798
**Reads uniquely mapped to RGAP7 (%)**	12,255,809 (18.8)	9,919,863 (25.8)
**Reads mapped uniquely to the rice mitochondrial genome (%)**	626,587 (0.96)	253,771 (0.66)
**Reads mapped uniquely to the rice chloroplast genome (%)**	102,730 (0.16)	36,915 (0.09)
**Total number of reads mapped to rice rRNA (%)**	41,744,369 (64.1)	26,875,340 (69.9)
**Reads mapped to SmR1 genome (%)**		24,263 (0.06)
**Total number of reads mapped (%)**	54,803,125 (84.2)	32,813,183 (85.3)
**Unmapped reads (%)**	10,296,195 (15.8)	5,650,615 (14.7)

Supplementary Table 4 – Genes involved in secondary metabolism and abiotic and biotic stresses modulated in rice roots colonised with *H. seropedicae*.

Gene ID	Gene product name/ description*	Fold change	P-value	P-adj
**Isoprenoid synthesis genes**				
LOC_Os07g09190.1	Transketolase, putative, expressed (MSU)<br>Similar to 1-deoxy-D-xylulose 5-phosphate synthase 2 precursor (DXS)<br>(RAPDB)	-5.9	1.30E-03	0.10
LOC_Os01g01710.1	1-deoxy-D-xylulose 5-phosphate reductoisomerase (DXR), chloroplast precursor,<br>putative, expressed (MSU)	-1.8	1.20E-03	0.09
LOC_Os01g58790.2	GHMP kinases ATP-binding protein, putative, expressed (MSU)<br>Similar to 4-diphosphocytidyl-2-C-methyl-D-erythritol kinase (CMK),<br>chloroplast precursor (RAPDB)	-1.5	0.03	0.59
LOC_Os02g39160.1	Hydroxymethylbutenyl 4-diphosphate synthase (HDS), putative, expressed (MSU)<br>Similar to Isoprenoid biosynthesis-like protein (RAPDB)	-1.3	0.04	0.64
LOC_Os03g52170.1	4-hydroxy-3-methylbut-2-enyl diphosphate reductase (HDR), putative, expressed (MSU)<br>Similar to Isopentenyl/dimethylallyl diphosphate synthase (RAPDB)	-1.5	0.04	0.65
LOC_Os07g39270.1	Polyprenyl synthetase, putative, expressed (MSU)	-2.6	6.70E-06	1.30E-03

	Similar to GGDP synthase (DGPS) (RAPDB)			
LOC_Os11g28530.2	Terpene synthase, putative, expressed (MSU)<br>Similar to Stemar-13-ene synthase (OsDTC2) (RAPDB)	-1.9	1.50E-03	0.11
**Isoflavonoid synthesis genes**				
LOC_Os12g02370.2	Chalcone-flavonone isomerase (CHI), putative, expressed (MSU)	-2.2	1.00E-04	0.01
LOC_Os06g44180.1	NAD dependent epimerase/dehydratase family protein, putative, expressed (MSU)<br>Similar to NADPH HC toxin reductase (RAPDB)	-2.5	0.04	0.63
LOC_Os03g03034.1	Flavonol synthase (FS)/flavanone 3-hydroxylase, putative, expressed (MSU)	-8.3	3.60E-07	1.00E-04
LOC_Os12g16280.1	Expressed protein (MSU)<br>Similar to Isoflavone reductase-like protein 1.(RAPDB)	-1.3	0.01	0.31
**Lignin synthesis genes**				
LOC_Os02g56700.1	Dehydrogenase, putative, expressed (MSU)<br>Similar to Cinnamoyl-CoA reductase (RAPDB)	2.76	8.10E-06	1.60E-03
**Secondary metabolism genes**				
LOC_Os01g63190.1	Laccase precursor protein, putative, expressed (MSU)	1.56	0.02	0.46
LOC_Os04g27070.1	Terpene synthase, putative, expressed (MSU)	-7.1	1.00E-09	4.00E-07
LOC_Os04g51660.1	Transferase family protein, putative, expressed (MSU)	-12.8	2.80E-03	0.16

LOC_Os08g14760.1	AMP-binding domain containing protein, expressed (MSU)<br>Similar to 4-coumarate--CoA ligase 1 (RAPDB)	1.48	0.03	0.57
LOC_Os09g31490.1	Reductase, putative, expressed (MSU)<br>NAD(P)-binding domain containing protein (RAPDB)	1.60	0.01	0.38
LOC_Os11g18366.2	Cycloartenol synthase, putative, expressed (MSU)	-2.7	1.00E-07	3.00E-05
**Cell wall**				
LOC_Os01g16770.1	Similar to Expansin-A11 (MSU)	2.6	0.02	0.53
LOC_Os04g44840.1	Glycosyl hydrolase family 3 protein, putative, expressed (MSU)<br>Similar to Beta-D-xylosidase (RAPDB)	2.0	1.35E-03	0.10
LOC_Os09g31270.1	Polygalacturonase, putative, expressed (MSU)	-2.4	6.95E-03	0.30
**Peroxidases**				
LOC_Os03g25300.1	Peroxidase precursor, putative, expressed (MSU)	2.2	5.87E-04	0.06
LOC_Os07g01410.1	Peroxidase precursor, putative, expressed (MSU)	2.8	2.86E-05	4.66E-03
**Protein degradation**				
LOC_Os01g19260.1	ATPase, putative, expressed (MSU)	2.1	1.94E-03	0.13
LOC_Os01g57082.1	Insulin-degrading enzyme, putative, expressed (MSU)<br>Peptidase M16, zinc-binding site domain containing protein (RAPDB)	-2.4	0.04	0.61
LOC_Os04g03796.1	OsSub37 - Putative Subtilisin homologue, expressed (MSU)<br>Peptidase S8, subtilisin-related domain containing protein (RAPDB)	-2.4	9.40E-13	4.65E-10
LOC_Os04g53620.1	Ubiquitin family protein, putative, expressed (MSU)	2.8	3.33E-16	2.00E-13

LOC_Os05g28730.1	Zinc finger, C3HC4 type domain containing protein, expressed (MSU)<br><br>Similar to PIT1 (RAPDB)	-2.2	0.01	0.38
LOC_Os05g48340.1	Proteasome-related, putative, expressed (MSU)<br><br>Armadillo-type fold domain containing protein (RAPDB)	-2.1	1.84E-03	0.12
LOC_Os06g05990.1	Zinc finger family protein, putative, expressed (MSU)	2.0	3.15E-05	4.98E-03
LOC_Os06g06600.1	OsFBX187 - F-box domain containing protein, expressed (MSU)	-2.2	5.29E-03	0.25
LOC_Os06g29310.1	ulp1 protease family protein, putative, expressed (MSU)<br><br>Peptidase C48, SUMO/Sentrin/Ubl1 family protein (RAPDB)	2.1	0.01	0.35
LOC_Os09g39070.1	Thiol protease SEN102 precursor, putative, expressed (MSU)	2.1	2.88E-04	0.03
**Redox**				
LOC_Os01g16152.1	Peroxiredoxin, putative, expressed (MSU)	-2.1	0.01	0.43
LOC_Os03g13160.1	Non-symbiotic hemoglobin 2, putative, expressed (MSU)	2.4	9.11E-04	0.08
LOC_Os03g12510.1	Non-symbiotic hemoglobin 2, putative, expressed (MSU)	104	5.58E-24	5.15E-21
LOC_Os07g44440.1	Peroxiredoxin, putative, expressed (MSU)	3.7	2.32E-05	3.90E-03
**Signalling**				
LOC_Os09g22450.1	Lipase, putative, expressed (MSU)<br><br>Lipase, class 3 family protein, gene name synonym: enhanced disease susceptibility 1(RAPDB)	2.0	0.01	0.36
LOC_Os02g01520.1	IQ calmodulin-binding motif family protein, putative, expressed (MSU)	2.2	0.03	0.60
LOC_Os02g30190.1	expressed protein (MSU)	2.0	0.04	0.64

LOC_Os03g19720.1	EF hand family protein, putative, expressed (MSU)<br><br>Similar to Hypersensitive reaction associated Ca2+-binding protein (RAPDB)	3.1	0.03	0.60
LOC_Os04g56430.1	Cysteine-rich receptor-like protein kinase, putative, expressed (MSU)	3.2	7.01E-05	0.01
LOC_Os05g47770.1	Serine/threonine-protein kinase At1g18390 precursor, putative, expressed (MSU)	2.0	0.01	0.36
LOC_Os07g03790.1	Lectin-like receptor kinase 7, putative, expressed (MSU)	-2.7	0.01	0.35
LOC_Os08g10320.1	SHR5-receptor-like kinase, putative, expressed (MSU)	-2.5	2.33E-05	3.90E-03
**Abiotic stress**				
LOC_Os01g67360.1	Methyltransferase, putative, expressed (MSU)<br><br>Similar to dehydration-responsive family protein (RAPDB)	-2.9	1.96E-03	0.13
LOC_Os06g29310.1	ulp1 protease family protein, putative, expressed (MSU)<br><br>Peptidase C48, SUMO/Sentrin/Ubl1 family protein (RAPDB)	2.1	0.01	0.35
LOC_Os08g08960.1	Cupin domain containing protein, expressed (MSU)<br><br>Similar to Germin-like protein precursor (RAPDB)	-2.7	3.72E-05	0.01
LOC_Os08g13440.1	Cupin domain containing protein, expressed (MSU)<br><br>Germin-like protein 8-12, Disease resistance (RAPDB)	-5.3	2.08E-04	0.03
**Regulation of transcription**				
LOC_Os11g02480.2	WRKY46, expressed (MSU)	-2.3	0.05	0.68
LOC_Os04g21950.1	WRKY51, expressed (MSU)	2.0	0.01	0.43
LOC_Os03g27090.1	MYB family transcription factor, putative, expressed (MSU)	3.6	5.93E-04	0.06

LOC_Os07g48570.1	Expressed protein (MSU)<br><br>Similar to Dof3 gene (Fragment) (RAPDB)	2.2	4.86E-03	0.24
**PR-protein**				
LOC_Os02g38392.1	NBS-LRR disease resistance protein, putative, expressed (MSU)	-2.7	3.28E-04	0.04
LOC_Os10g25870.1	Dirigent, putative, expressed (MSU)<br><br>Plant disease resistance response protein family protein (RAPDB)	-2.5	0.04	0.65
LOC_Os11g07680.1	Dirigent, putative, expressed (MSU)<br><br>Conserved hypothetical protein (RAPDB)	-3.4	4.45E-03	0.23

* Gene product name/description from the both rice database were used: MSU (Rice Genome Annotation Project) and RAPDB (The Rice Annotation Project).

Supplementary Table 6 – Oligonucleotides used in this research

Gene Product Name (MSU)	Locus name	Abbreviations	Sequence	[nM] of each primer	Efficiency (threshould 0.2)
Actin 1	AK100267	Act	F 5’CAGCCACACTGTCCCCATCTA3’<br>R 5’AGCAAGGTCGAGACGAAGGA3’	200	1.892
Tubulin beta-2 chain (Beta-2 tubulin).	LOC_Os01g59150	Tub	F 5’GCTGACCACACCTAGCTTTGG3’<br>R 5’AGGGAACCTTAGGCAGCATGT3’	200	1.895
Conserved hypothetical protein	LOC_Os06g48970.1	Hypot	F 5’ATGGACTGAAAAGGCTGAGTTGA3’<br>R 5’GCACCCTGATTGGTGGACTT3’	200	1.882
Tubulin/FtsZ domain containing protein, putative, expressed	LOC_Os07g38730.1	Tub/FtsZ	F 5’ ATGAGCAGCTTTCTGTGCCT 3’<br>R 5’ CGGTACATCAAGCAGCAAGC 3’	200	2.01
Eukaryotic translation initiation factor 3 subunit C, putative, expressed	LOC_Os07g03230.2<br>Os07t0124500-01	Os07g03230	F 5 GTGCAACAAAGTCGATACCATGAA 3’<br>R 5’ ACAAATCAAGTGAGTGGCTTCCA 3	400	2.03
Glutamine synthetase, catalytic domain containing protein, expressed	LOC_Os03g12290.1<br>Os03t0223400-01	GS	F 5’ CAACCTCACCGACCTCGTTAA 3’<br>R 5’ CCTTTCACAGTCCTCGCTTTG 3’	400	1.94
Wall-associated receptor kinase-like 22 precursor, putative, Expressed	LOC_Os10g07556.1<br>Os10t0162844-00	WARK22	F 5’ ATCAAGTGAGCAAAGGGAACT 3’<br>R 5’ GCGATGATCAATGGCGTTGT 3’	400	1.99
Integral membrane protein, putative, expressed (MSU); Vacuolar membrane transporter, Fe and Zn translocation between flag leaves and seeds (RAPDB)	LOC_Os09g23300.1<br>Os09t0396900-02	Int_Memb	F 5’ GCGGAGATCGCGGACATA 3’<br>R 5’ TCCCAACTCAAACTTCATCATGA 3’	400	1.81
Major facilitator superfamily antiporter, putative, expressed (TOM1)	LOC_Os11g04020.1<br>Os11g0134900	Tom1	F 5’ GCCCAAGAACGCCAAAATGA 3’<br>R 5’ GGCTTGAAGGTCAACGCAAG 3’	400	2.09
Non symbiotic hemoglobin 2	LOC_Os03g12510.1<br>Os03t0226200-01	rHb2	F 5’ GCTCTCGTGGAGGGAAACAA 3’<br>R 5’ CTTCATGATGGCCCACGACT 3’	400	2.06
Metal cation transporter, putative, expressed (OsIRT2)	LOC_Os03g46454.1<br>Os03t0667300-00	OsITR2	F 5’ CACCAAGGCGCAGCTTCT 3’<br>R 5’ CGGATCGTGCACACGTTCT 3’	400	2.07
Flavonol synthase/flavanone 3-hydroxylase, putative, expressed	LOC_Os03g03034<br>Os03t0122300-01	Flavo_Synt	F 5’ CCCGCTTCACCGCTACCT 3’<br>R 5’ TCGGATATCGCTCCGTACAGT 3’	400	1.97
Iron-phytosiderophore transporter	LOC_Os02g43410<br>Os02t0650300-01	YSL	F 5’ CCACACAGCCCAAGGAGATAA 3’<br>R 5’ TGCCTCCCGTGTAGAACCAT 3’	400	1.98
Metal transporter Nramp6, putative, expressed	LOC_Os07g15460<br>Os07t0258400-03	Nramp6	F 5’ GAAAGGAGCCAGCATGGAAA 3’<br>R 5’ ATTTGTGATTGGCACCAGCTT 3’	400	2.00
Thionin-like peptide, putative, expressed	LOC_Os07g24830.1<br>Os07g0429700	Thionin7	F 5’ TGGTCATCTTGAGCCTCACTGT 3’<br>R 5’ GGAGCCACCAGCAGCAATAC 3’	200	1.90
Pathogenesis-related Bet v I family protein, putative, expressed (MSU);<br>Similar to Probenazole-inducible protein PBZ1 (RAPDB)	LOC_Os12g36840.1<br>Os12t0555100-01	PBZ1	F 5’ GGCACCGTTGTCATTCTGAA 3’<br>R 5’ GTGCCACCACACGTGTCTTG 3’	400	1.82
Conserved hypothetical protein.	Os06t0513862-01	Thionin1	F GCTGAGGAATCGGAGGTAGTTG<br>R TTCTCATGGTGCTGCACACA	200	1.93
